# New-Onset Paroxysmal Atrial Fibrillation in a Healthy Male With Post-COVID-19 Painless Thyroiditis: A Case Report

**DOI:** 10.7759/cureus.66288

**Published:** 2024-08-06

**Authors:** Valerie Chavez-Flores, Andro Sharobiem, Steven Kim, Michael Kang, Tommy Y Kim

**Affiliations:** 1 Emergency Medicine, HCA Healthcare, Riverside Community Hospital, Riverside, USA; 2 Internal Medicine, HCA Healthcare, Riverside Community Hospital, Riverside, USA

**Keywords:** emergency, cardiac arrythmia, paroxsymal atrial fibrillation, painless thyroiditis, covid-19

## Abstract

Painless thyroiditis is a variant of thyroiditis without the typical neck pain and is otherwise similar to subacute thyroiditis, which is a known post-viral condition and has been associated with coronavirus disease 2019 (COVID-19) infections. While it is usually self-limiting, it can lead to thyrotoxicosis that can predispose individuals to cardiac dysrhythmias, including atrial fibrillation. There has been a clear association between COVID-19 and subacute thyroiditis with a few case reports describing atrial fibrillation. We present a case of a healthy patient with new-onset atrial fibrillation secondary to painless thyroiditis. This report highlights the rare entity of painless thyroiditis leading to atrial fibrillation with rapid ventricular response in a patient who recently recovered from COVID-19. Physicians should consider the potential association of painless thyroiditis in patients with new-onset atrial fibrillation who recently recovered from COVID-19.

## Introduction

Thyroiditis, specifically subacute thyroiditis, is defined as a self-limiting inflammatory disorder of the thyroid gland, often triggered by recent viral infections [1]. During the coronavirus disease 2019 (COVID-19) pandemic, more evidence associating subacute thyroiditis with SARS-CoV-2 was uncovered. The typical clinical manifestations of a painful thyroid gland, malaise, anorexia, palpitations, and fever occur approximately 28 days after a viral infection [2,3]. Painless thyroiditis is a variant of thyroiditis without the associated clinical manifestations such as neck pain and is otherwise similar to subacute thyroiditis [4]. To our knowledge, there is a scarcity of reported cases of painless thyroiditis after recovery from COVID-19 [5-7]. Thyroid inflammation and dysfunction can predispose individuals to cardiac dysrhythmias, including atrial fibrillation. Atrial fibrillation has been described in cases of post-COVID-19 subacute thyroiditis, but cases of atrial fibrillation associated with painless thyroiditis are lacking. We discuss a rare case of a healthy patient with post-COVID-19 painless thyroiditis who presented with new-onset paroxysmal atrial fibrillation.

## Case presentation

**Figure 1:**
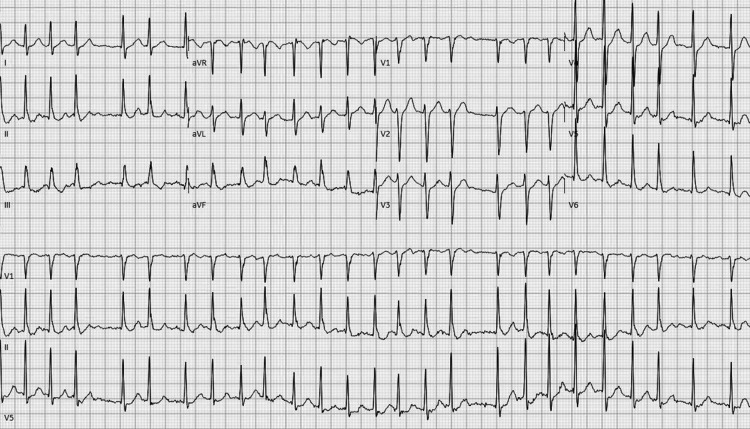
Electrocardiogram showing atrial fibrillation with rapid ventricular response

A 51-year-old healthy male with a history of recently resolved COVID-19 infection presented to the emergency department (ED) with two hours of acute-onset palpitations and shortness of breath. In the ED, his initial vital signs showed a temperature of 37.4 ˚C, heart rate of 140 beats per minute, respiratory rate of 18 breaths per minute, blood pressure of 188/81 mmHg, and an oxygen saturation of 97%. The patient’s electrocardiogram (ECG) revealed atrial fibrillation with rapid ventricular response with a heart rate of 154 (Figure 1).

The patient had tested positive for COVID-19 one month prior with symptoms of skin hyperesthesia, myalgia, mild cough, and low-grade fevers and had complete resolution of symptoms. On further review of systems, the patient denied any previous history of atrial fibrillation, thyroid disease, weight loss, diaphoresis, chest pain, neck swelling, or neck pain. On examination, the patient had an irregular heart rhythm with no obvious neck swelling or tenderness. Laboratory testing was significant for high thyroxine (T4) of 14.7 mcg/dL (normal range: 4.5 - 12.1 mcg/dL), high free T4 1.7 ng/dL (normal range: 0.76 - 1.46 ng/dL), low thyroid stimulating hormone (TSH) of 0.008 uIU/mL (normal range: 0.358 - 3.74 uIU/mL), and a normal thyroid peroxidase antibody 16 IU/mL (normal range: 0 - 34 IU/mL). Other laboratory tests were found to be negative, including a comprehensive metabolic panel, D-dimer, and troponin I (Table 1).

**Table 1:** Initial laboratory testing consistent with hyperthyroidism

Test	Result	Normal values
Sodium	143 mmol/L	136 – 145 mmol/L
Potassium	4.2 mmol/L	3.5 – 5.1 mmol/L
Chloride	114 mmol/L	98 – 107 mmol/L
Carbon dioxide	25 mmol/L	21 – 32 mmol/L
Blood urea nitrogen	12 mg/dL	7 – 18 mg/dL
Creatinine	0.9 mg/dL	0.7 – 1.3 mg/dL
Glucose	129 mg/dL	74 – 106 mg/dL
Total bilirubin	0.7 mg/dL	0.2 – 1 mg/dL
Aspartate transferase	25 U/L	15 – 37 U/L
Alanine transaminase	68 U/L	12 – 78 U/L
Alkaline phosphatase	84 U/L	45 – 117 U/L
D-dimer	293 ng/mL	<500 ng/mL
High-sensitivity troponin I	11 ng/L	3 – 78 ng/L
Thyroxine	14.7 mcg/dL	4.5 – 12.1 mcg/dL
Free thyroxine	1.7 ng/dL	0.76 – 1.46 ng/dL
Thyroid-stimulating hormone	0.008 uIU/mL	0.358 – 3.74 uIU/mL
Thyroid peroxidase antibody	16 IU/mL	0 – 34 IU/mL

**Figure 2:**
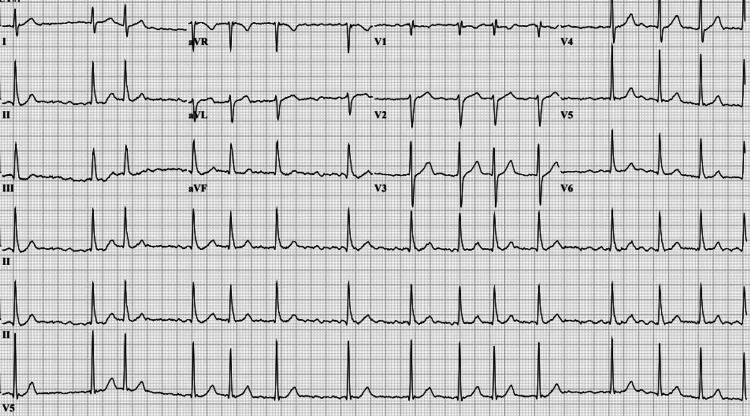
Electrocardiogram showing persistent atrial fibrillation with improved heart rate

The patient was deemed to be in a thyroid storm based on his Burch-Wartofsky score [8] of 45, heart rate greater than 140 beats/minute (25 points), presence of atrial fibrillation (10 points), and the precipitating event of COVID-19 (10 points). He was treated with diltiazem 25 mg intravenously, which improved the heart rate; however, he remained in persistent atrial fibrillation (Figure 2).

**Figure 3:**
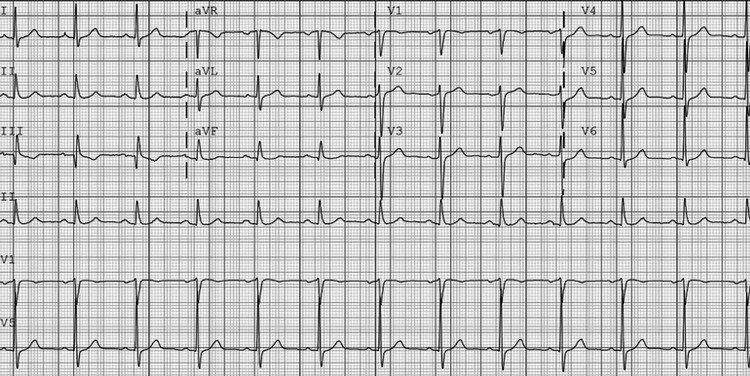
Electrocardiogram showing normal sinus rhythm after spontaneous cardioversion

The patient was admitted for new-onset atrial fibrillation and thyroid storm with plans for pharmacological cardioversion with amiodarone. Soon after the admission, the patient spontaneously self-converted to a normal sinus rhythm with the resolution of his previous symptoms (Figure 3).

Thyroid ultrasound revealed a nonspecific diffusely heterogeneous echogenicity of the thyroid gland compatible with thyroiditis, and a cardiac echocardiogram was unremarkable except for trivial mitral valve regurgitation. The patient was discharged on a beta-blocker for rate control and a nonsteroidal anti-inflammatory medication for thyroiditis.

At the one-month follow-up, the patient remained asymptomatic, and repeat laboratory testing revealed a persistently elevated T4, an improving TSH, and normal thyroglobulin and thyroid peroxidase antibodies (Table 2).

**Table 2:** Thyroid testing at the one-month follow-up

Test	Result	Normal values
Thyroxine	13.9 mcg/dL	4.9 – 10.5 mcg/dL
Free thyroxine index	4.9	1.4 – 3.8
Thyroid-stimulating hormone	0.02 uIU/mL	0.40 – 4.50 uIU/mL
Thyroid peroxidase antibody	2 IU/mL	<9 IU/mL
Thyroglobulin antibody	1 IU/mL	≤1 IU/mL

At the two-month follow-up, repeat laboratory tests revealed normalizing T4 and an improving TSH level. The patient remained asymptomatic at the two-month follow-up (Table 3).

**Table 3:** Thyroid testing at the two-month follow-up

Test	Result	Normal values
Thyroxine	10.5 mcg/dL	4.9 – 10.5 mcg/dL
Free thyroxine index	3.3	1.4 – 3.8
Thyroid-stimulating hormone	0.01 uIU/mL	0.40 – 4.50 uIU/mL
Thyroid peroxidase antibody	2 IU/mL	<9 IU/mL
Thyroglobulin antibody	1 IU/mL	≤1 IU/mL

## Discussion

Since the emergence of the COVID-19 pandemic, several cases of subacute thyroiditis have been reported in the medical literature [4]. Ashrafi et al. estimated that thyrotoxicosis was the second most common post-COVID-19 thyroid disease with a prevalence of about 10% (95% CI: 4 - 16%) of all thyroid-related illnesses [9]. In an international population-based propensity score-matched analysis of patients diagnosed with COVID-19 and those with COVID-19-like symptoms who tested negative, Huang et al. found the COVID-19 group with an increased risk [hazard ratio: 2.10 (95% CI: 1.92 - 2.29)] for thyrotoxicosis [10]. The symptoms associated with subacute thyroiditis from COVID-19 are similar to other known associated viral etiologies and include neck pain, goiter development, and other thyrotoxic symptoms including palpitations and fever [11].

Unique to our patient was the patient’s presentation with atrial fibrillation in the setting of painless thyroiditis. Several case reports with atrial fibrillation involve patients with post-COVID-19 painless thyroiditis who were later diagnosed with Graves’ disease [12,13]. Other case reports with patients presenting with atrial fibrillation secondary to post-COVID-19 subacute thyroiditis reported signs and symptoms consistent with thyroiditis with neck pain and tenderness [1]. Hashemipour et al. showed that 6.9% of patients hospitalized with COVID-19 had subclinical/overt thyrotoxicosis with atrial fibrillation in 37.5% of this group, but they failed to differentiate patients with subclinical and overt disease [14]. Lui et al. noted that post-COVID-19 painless thyroiditis differed from subacute thyroiditis in several aspects; painless thyroiditis presented sooner after recovery from COVID-19 and was more prevalent in patients with severe symptoms of COVID-19 [4]. Our case was different as the patient's presentation was similar to subacute thyroiditis with mild symptoms of COVID-19 and atrial fibrillation occurring one month after recovery from COVID-19.

While the exact pathogenesis of this condition remains ill-defined, it is speculated that viral infections induce an autoimmune response leading to thyroid gland inflammation. Ashrafi et al. speculated that SARS-COV-2 affects the thyroid either directly via direct viral effects or indirectly through immune system dysregulation; however, more research is necessary to gather conclusive evidence [9]. Ganie et al. have postulated that the thyroid gland may be vulnerable to SARS-COV-2 due to the abundance of angiotensin-converting enzyme 2 receptors in the thyroid parenchyma, which may affect the thyroid function [2].

The treatment for painless thyroiditis is similar to that of subacute thyroiditis, consisting of conservative medical therapy, including nonsteroidal anti-inflammatory drugs (NSAIDs) for minor symptoms and steroids and beta-blockers for moderate symptoms. Ultimately, the course of both painless and subacute thyroiditis seems to be self-limiting [2].

## Conclusions

This case highlights the potential association between painless thyroiditis and atrial fibrillation in patients recovering from COVID-19. Most notable in this case was the presentation of painless thyroiditis with no symptoms of neck pain or swelling. Painless thyroiditis should be considered in patients with a recent history of COVID-19 who present with new-onset atrial fibrillation. Timely recognition and management of both thyroiditis and dysrhythmias are critical for preventing complications and ensuring favorable outcomes. Further research is warranted to clarify the association and devise management strategies for thyroid-related complications in COVID-19 survivors.
